# Evaluating the Effect of Active Charcoal-Containing Toothpaste on Color Change, Microhardness, and Surface Roughness of Tooth Enamel and Resin Composite Restorative Materials

**DOI:** 10.1155/2023/6736623

**Published:** 2023-05-09

**Authors:** Ali Forouzanfar, Pouria Hasanpour, Yasaman Yazdandoust, Hosein Bagheri, Hamideh Sadat Mohammadipour

**Affiliations:** ^1^Dental Research Center, School of Dentistry, Mashhad University of Medical Sciences, Mashhad, Iran; ^2^School of Dentistry, Mashhad University of Medical Sciences, Mashhad, Iran; ^3^Dental Materials Research Center, School of Dentistry, Mashhad University of Medical Sciences, Mashhad, Iran; ^4^Department of Restorative and Cosmetic Dentistry, School of Dentistry, Mashhad University of Medical Sciences, Mashhad, Iran

## Abstract

**Methods:**

Thirty-six bovine incisors and resin composite samples were prepared, stained with black tea, and then randomly divided into two groups. The samples were brushed with a charcoal-containing toothpaste (Colgate® MAX WHITE) and daily toothpaste (Colgate® Max Fresh) for 10,000 cycles. Before and after brushing cycles, color variables (*ΔL*, *Δa*, *Δb*), total color change (*ΔE*), plus Vickers microhardness were evaluated. Two samples of each group were prepared for surface roughness assessment via atomic force microscope. Data were analyzed by Shapiro–Wilk, Independent sample *t*-test and Mann–Whitney *U* tests.

**Results:**

According to the obtained results, *ΔE* and *ΔL* were significantly higher whereas *Δa* and *Δb* were noticeably lower in charcoal-containing toothpaste group in comparison with daily toothpaste in both composite and enamel samples. The microhardness of samples brushed with Colgate® MAX WHITE was significantly higher than that of Colgate® Max Fresh in enamel (*P* = 0.04), whereas no significant difference was found in composite resin samples (*P* = 0.23). Colgate® MAX WHITE enhanced the roughness of both enamel and composite surfaces.

**Conclusion:**

The charcoal-containing toothpaste could improve the color of both enamel and resin composite with no negative effect on microhardness. Nevertheless, its adverse roughening effect should be considered occasionally on composite restorations.

## 1. Introduction

Tooth discoloration is a major reason for the patients' referral to dentists. Tooth staining may arise from intrinsic and extrinsic sources [1]. Intrinsic stain originates from the deeper tooth surfaces and is caused by hereditary disorders, medications, fluorosis, and trauma. Extrinsic stains are located on the tooth surfaces and may be related to poor oral hygiene, smoking habit, and consumption of chromogenic food such as coffee and tea [2].

Multiple methods have been suggested to improve tooth color, depending on the stain type, extent, and depth of discoloration. They vary from very conservative treatments including scaling and polishing, bleaching, micro- and macroabrasions to more aggressive treatments such as ceramic laminates and crowns [3]. The application of the over-the-counter whitening products including powders and toothpastes may be more favorable for some patients [4]. Most tooth-whitening toothpastes remove extrinsic stains through abrasive particles such as the hydrated silica, calcium carbonate, dicalcium phosphate dihydrate, alumina, perlite, calcium pyrophosphate, and hexametaphosphate, whereas others make the teeth whiter and lighter and can even change the dentin's inherent color through hydrogen peroxide and other oxidizing agents [5]. New whitening systems containing natural agents have shown promising results in tooth whitening [3].

Activated charcoal is a black, odorless, and tasteless powder obtained from the removal of water and other volatile constituents from carbon-based materials [6]. This material has several applications in medical fields such as for pain relief, reducing swelling and burning, decreasing cholesterol levels, and preventing cholestasis in pregnancy [6–8]. It has also shown great antibacterial and anti-inflammatory properties [7, 9, 10]. The nanosized pores of charcoal enhance the surface area and facilitate the ion exchange in the mouth [11, 12]. According to the manufacturers' claims, it has capacity of adsorbing and removing pigments, chromophores, and stains responsible for the color change of teeth [6–9], which was more considered by the juvenile population [13].

Despite favorable properties, there are concerns regarding the adverse effects of charcoal-containing toothpastes on dental and oral health [7, 14]. The abrasive toothpastes can roughen hard tissue and restorations, damage the soft tissue, cause gingival recession, cervical abrasion, and dentin hypersensitivity [15, 16]. The average particle size of toothpaste containing activated charcoal is larger than that of plain toothpaste [17], with the star-shaped of coal particles also increasing the surface wear. The roughened surface is prone to retention of plaque, stains, and food debris, as well as increases the risk of caries and periodontal inflammation [18, 19]. They may also reduce the mechanical properties of enamel such as surface hardness [20]. Furthermore, the retention of charcoal particles in the gingival sulcus and formation of surface defects such as deep pits plus fissures on restorations result in compromised esthetic of teeth colored restorations [21]. Farghal and Elkafrawy [9] indicated the adverse effect of activated charcoal on surface roughness and microhardness of microfilled and nanohybrid composite resins. Thus, more evidence is required to assess the performance and safety of these products, and relying on only manufacturers' claims is not logical.

This study aimed to evaluate color change, surface hardness, and roughness of a commercial charcoal-containing toothpaste (Colgate® MAX WHITE) on tooth enamel and composite resin and to compare it with nonwhitening and daily toothpaste (Colgate® Max Fresh). The tested null hypotheses were as follows: charcoal-based whitening toothpaste (1) would not lead to tooth whitening and there would be no significant difference with regular toothpaste in terms of tooth whitening, (2) would not affect the microhardness, and finally (3) would not impact the surface roughness of bovine incisors and resin composites after 10,000 cycles of brushing.

## 2. Materials and Methods

### 2.1. Study Design

This *in vitro* study was conducted at the Department of Esthetic and Restorative Dentistry (Mashhad University of Medical Sciences, Mashhad, Iran). The study protocol was approved by the local ethics committee of Mashhad University of Medical Sciences, Iran (098. IR.MUMS.DENTISTRY.REC.1398).

### 2.2. Sample Size Calculation

The power of the sample size was calculated by G Power software (version 3.1, Heinrich Heine Dusseldorf University, Dusseldorf, Germany) with a 95% confidence interval and 80% power. Based on Khamvardi et al. [22] study, examining the effect of two whitening toothpastes on microhardness of enamel and composite, the sample size for each group in this study was calculated to be 18 (effect size = 0.41). Also, to evaluate the color, according to Karadas and Duymus [23] study, the sample size of 15 samples was estimated in each group (effect size = 0.76). Hence, 18 specimens per group were calculated as the minimum sample size.

### 2.3. Preparation of Enamel Samples

Thirty-six sound and intact bovine incisors were collected. The tissue debris was removed by a scaler (LM-Dental, Parainen, Finland), the teeth were cleaned under running tap water, and then examined by a stereomicroscope (Dino lite Pro, Anmo Electronic, New Taipei City, Taiwan) under magnification ×10 to exclude those with caries, erosion, enamel cracks, deep grooves, fracture, or sever discolorations. The teeth were kept in 0.1% thymol solution for 1 week and placed in the normal saline solution until the initiation of the experiment [22, 24]. To create a smoother enamel surface, 1,000 and 1,500 grit sandpapers (Starcke GmbH & Co. KG, Melle, Germany) were used under water flow. To prevent the penetration of black tea solution into the pulp space and dentinal tubules, a wax was placed into the root apex beforehand.

### 2.4. Preparation of Resin Composite Samples

Thirty-six disk-shaped resin composite specimens with an inner diameter of 10 and 2 mm thickness were fabricated. Uncured composite (Filtek Z250, 3M Co., St. Paul, Minnesota, USA) with A2 shade was packed into a custom-made ring mold sandwiched between two clear strips and two glass plates. This negative mold was made through pressing a metal object into a silicon material (Speedex, Coltene, Altstätten, Liechtenstein). The specimens were polymerized by a light-curing device (Bluephase C8, Ivoclar Vivadent, Schaan, Liechtenstein) with a minimum intensity of 650 mW/cm^2^ for 20 s. The intensity of the light source was checked using a light meter (Apoza, Apoza Enterprise Co. Ltd., Taiwan). Finishing and polishing were performed using Sof-Lex discs (3M Co., St. Paul, Minnesota, USA) according to the manufacturer's recommendation and sequences. The prepared specimens were pressed into a silicone material (Speedex, Coltene, Altstätten, Liechtenstein) for simple handling during finishing and polishing procedures. For each disc, five movements in one direction were used in a rough to soft sequence. The specimens were removed carefully from the mold, then immersed in distilled water, and eventually stored inside an incubator (Fine Tech, Shin Saeng, Gyeonggi-do, South Korea) at 37°C and 100% humidity for 24hr to simulate mouth temperature and complete polymerization.

### 2.5. Staining Process

To simulate tooth staining, black tea solution (Golestan, Tehran, Iran) was prepared through immersing five tea bags into a container containing 500 ml of boiling water. After waiting for 10 min to achieve drinking temperature, the specimens were immersed into the black tea solution and then stored inside an incubator (Fine Tech, Shin Saeng, Gyeonggi-do, South Korea) for 10 days. Following the simulated staining process, the tooth crowns were separated from the roots and the crowns were embedded in self-cured acrylic resin blocks (Acropars, Marlic Co., Tehran, Iran) leaving the buccal enamel surfaces exposed and were parallel to the horizon and upper surface of the acrylic resin block. The same manner was applied for the composite resin samples.

### 2.6. Brushing Process

The stained bovine incisors and resin composite discs were randomly divided into two groups (*n* = 18) according to the toothpastes used:

  Group 1: The specimens were subjected to brushing with charcoal-containing toothpaste (Colgate® MAX WHITE Charcoal, Colgate-Palmolive Company, NY, USA).
  Group 2: The samples were subjected to brushing with daily and noncharcoal-containing toothpaste (Colgate® Max Fresh - Cool Mint Flavor, Colgate-Palmolive Company, NY, USA).

The compositions of the two toothpastes applied in this study are presented in Table 1.

The brushing process was performed by a brushing machine (Nemo, Tehran, Iran). This machine had four containers and arms applying horizontal brush strokes and allowing brushing of all four specimens simultaneously. The specimens were fixed in place with screws to limit the movement during brushing cycles. The brushing head of each toothbrush (Oral B, Acumen Houseware Industry Co., Binh Doung, Vietnam) was removed from the handle and fixed in a machine, in which the bristles of the brush heads were positioned vertically to the surface of the specimens. The filaments of the brushes were soft. One brush head was used for every six teeth. The brushing load was 45 N with a linear movement pattern in a horizontally one-way direction with a travel length of 4 mm and a speed of 99 mm/s. Specimens were individually subjected to 10,000 mechanical brushing cycles, which is equal to 12 months of brushing in a regular oral hygiene practice [25].

Slurry of water and toothpaste with a ratio of 3 : 1 (by weight) was prepared and purred in the brushing container of the brushing machine [10, 24, 26]. Specifically, 1 g of dentifrice was mixed with 3 ml of water and was poured above the samples to simulate brushing in an aqueous environment. Tooth brushing was performed in a humid environment when an operator loaded the toothbrush heads with a new mixture. After brushing, the specimens were washed, dried, and submitted to the study measurements.

Specimens were evaluated for color stability, surface microhardness, and roughness at two intervals: at baseline and after brushing with two toothpastes. Along the testing intervals for all groups, specimens were maintained in distilled water at 37°C in the incubator (Fine Tech, Shin Saeng, Gyeonggi-do, South Korea).

### 2.7. Color Measurements

The color change was measured according to the Commission Internationale de l'Eclairage L^*∗*^a^*∗*^b^*∗*^ values under the standard D65 illumination by a colorimeter (Chroma meter, Konica Minolt, Japan). Colorimetry was applied under the standard conditions to avoid subjective misinterpreted evaluation. To eliminate ambient light and equivalent tooth shade area for all samples, a silicon index was made from impression material (Speedex, Coltene, Altstätten, Liechtenstein). The *L*^*∗*^ value represents the degree of lightness of a specimen, varying from black (0) to white (100). The a^*∗*^ values red (+*a*) to green (*–a*) and b^*∗*^ values yellow (+*b*) to blue (*–b*) in the specimens. All measurements were repeated three times and the average of the three readings was calculated. The total color difference between baseline measurements and after the brushing process was calculated through applying the Hunters equation [24]:

(1) $$\begin{array}{c} \Delta E=\sqrt{{\left(a2-a1\right)}^{2}+{\left(b2-b1\right)}^{2}+{\left(L2-L1\right)}^{2}} \end{array}$$

### 2.8. Vickers Microhardness Test

The degree of microhardness of specimens was evaluated by microhardness tester (KOOPA PAZHOOHESH, Iran, Model: MH3). A pyramidal diamond indenter was used on each specimen to form a square indent, using a 100 N load at room temperature. The indenter was pressed into the sample with an accurately controlled test force maintained for 10 s. The size of the indent was determined optically through measuring the two diagonals of the square indent. The Vickers hardness number is a function of the test force divided by the surface area of the indent. The average of the three diagonals at a distance of 100 *μ*m from each other was employed to calculate the Vickers microhardness values [24].

### 2.9. Surface Roughness

Two samples from each group were used to assess the surface roughness. An atomic force microscope (Ara Research, Tehran, Iran) was applied in this study to evaluate the surface roughness of the specimens. The samples were placed on the stage and manually adjusted to project an image onto the monitor screen. The device would detect the surface roughness by measuring the depth of a single valley with the height of the surrounding peaks using a pointed, cone-shaped probe. Each sample was scanned at three intervals and averaged accordingly to determine the roughness (Ra) value [17, 24].

### 2.10. Statistical Analysis

Descriptive statistics for each group were reported as means and standard deviations. Data were statistically analyzed by SPSS statistical package version 22 (Released 2011; SPSS Inc., IBM Corp., Armonk, New York, USA). The Shapiro–Wilk test was used to verify the normality of distribution. The data were statistically analyzed by the Independent sample *t*-test and the Mann–Whitney *U* test to determine any significant difference in surface roughness and hardness for each normal and abnormal distribution of data, respectively. The significance level was set at 0.05.

## 3. Results

### 3.1. Color Variables

Mean values and standard deviations of color change and variables are reported in Table 2. According to Shapiro–Wilk analysis, the data of both enamel and composite groups revealed normal distribution (*P* > 0.05), except in *ΔL* of tooth samples and *Δa* in composite samples submitted to Colgate® Max Fresh toothpaste (*P* < 0.05).

Based on Mann–Whitney *U* and Independent sample *t*-test, the mean value of *ΔL* was significantly greater in both enamel and composite samples brushed with the charcoal-containing toothpaste compared with the control toothpaste (*P* = 0.001 and 0.021, respectively). The resin composite and enamel specimens treated with Colgate® MAX WHITE toothpaste indicated significantly lower *Δa* and *Δb* in comparison with those brushed with Colgate® Max Fresh toothpaste (*P* < 0.05).

### 3.2. Color Change (*ΔE*)

According to the Shapiro–Wilk analysis, the normal distribution of all groups was confirmed (*P* > 0.05), except in the study group in which Colgate® Max Fresh toothpaste was used on the enamel samples (*P* < 0.05).

Based on Independent sample *t*-test and Mann–Whitney *U* tests, the charcoal-containing toothpaste showed significantly higher *ΔE* mean values in both composite and enamel specimens compared with the control toothpaste, respectively (*P* ≤ 0.001) (Table 3).

### 3.3. Microhardness and Surface Roughness

**Figure 1:**
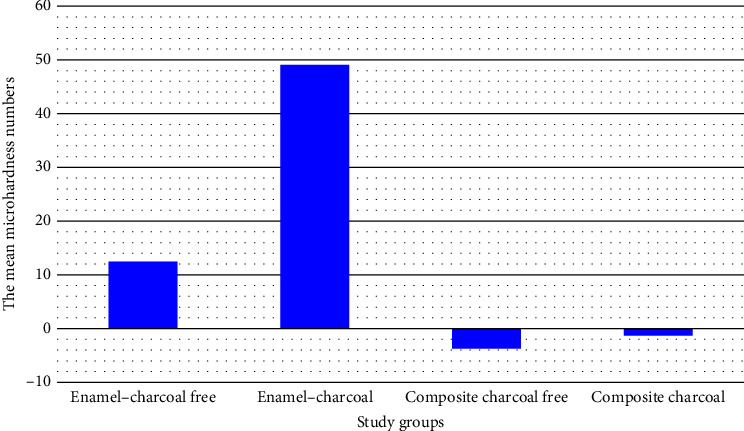
The mean microhardness numbers of enamel and resin composite samples after brushing with the evaluated charcoal-free and charcoal-containing toothpastes.

The results of microhardness evaluation are presented in Table 4 and Figure 1. The Shapiro–Wilk analysis revealed normal distribution of all data (*P* > 0.05). There was no significant difference between experimental and control groups in composite samples (*P* = 0.23). In enamel samples, the mean value of microhardness in charcoal-containing toothpaste group was noticeably greater than in the control toothpaste (*P* = 0.04).

**Figure 2:**
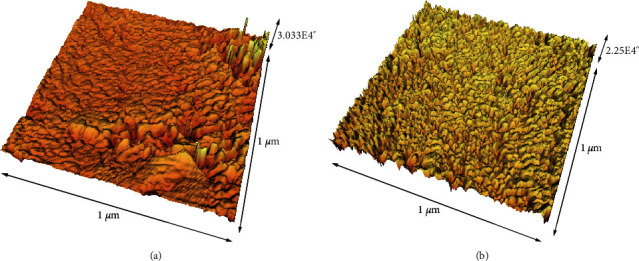
The 3D image of enamel samples before (a) and after (b) brushing with the charcoal-containing toothpaste (Colgate® MAX WHITE).

**Figure 3:**
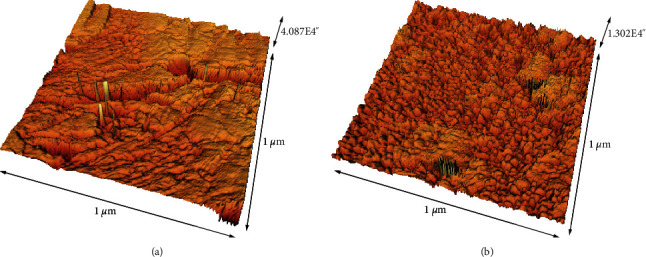
The 3D image of resin composite samples before (a) and after (b) brushing with the charcoal-containing toothpaste (Colgate® MAX WHITE).

The mean roughness values of composite and enamel samples are outlined in Table 5. As presented in Figures 2 and 3, the surface roughness of both enamel and composite samples was enhanced after brushing with charcoal-containing toothpaste. However, due to lack of specimens' number, the statistical analysis could not be done on these data.

## 4. Discussion

This study aimed to assess the effect of a toothpaste containing activated charcoal on color, microhardness, and surface roughness of bovine enamel and composite specimens compared to regular toothpaste. Since the charcoal-containing toothpaste indicated significantly lower *Δa* and *Δb* as well as higher *ΔE* and *ΔL* in both resin composite and enamel plus greater enamel microhardness compared with regular toothpaste, all hypotheses of this research were rejected.

Since bovine teeth have larger surfaces, fewer defects, more accessibility as well as similar structure to human teeth, they were used in this study for color and hardness assessments. Based a previous clinical study and considering a person's average brushing which ranges from 25 to 30 cycles per day, during 6 months 4562–5475 cycles may be done [25]. Thus, in this study, 10,000 cycles were used to simulate about 12 months of brushing where the brushing machine was used to standardize the brushing forces, duration, and speed on all specimens.

In this study, the *ΔE* parameter was utilized to compare the color change after applying the toothpastes. It describes color change using three dimensions of *L*, *a*, and *b*. The obtained results indicated significantly greater mean values of *ΔL* and *ΔE* plus lower values of *Δa* and *Δb* in both enamel and composite specimens after using charcoal-containing toothpaste. Since *ΔE* values above 3.3 are considered as the clinical threshold detected by the human eyes [27], the color change in enamel groups for both toothpastes was clinically obvious (17.83 in experimental toothpaste and 7.49 in control one). The higher overall color change of the charcoal-containing toothpaste treated group in enamel samples confirmed the better performance of this toothpaste in enamel as compared to the composite specimens. The obtained value of *ΔE* in this study was greater than the one found in a similar study performed by Ghajari et al. [28], who reported an average of three for the same Colgate® MAX WHITE. The difference may probably relate to the higher brushing cycles in this study which was more effective in removing pigments, as well as to the difference in human and bovine teeth. Although the wear caused by the bristles of the toothbrush can mechanically remove the surface pigments created through consuming tea and other colored food, since both control and experimental groups used the same brushes in the current study, the achieved results can solely be related to the whitening effects of the charcoal-containing toothpaste. The activated charcoal can improve color and whiten teeth through two main mechanisms: mild abrasion and absorption of stains. The latter was constructed on adsorption capacity and greatly porous structure with high-reaching surface area which can adsorb pigments, chromophores, or stains responsible for discoloration [24].

Although the whitening effect of charcoal was confirmed in this study, some authors have questioned the whitening efficacy of charcoal toothpastes [29] or even have rejected it [24, 30]. For instance, Febriani et al. [31] showed activated charcoal could absorb positively charged pigments, or it may improve tooth color as efficiently as hydrogen peroxide-containing toothpastes [26], whereas some authors reported no color change after using charcoal-containing toothpaste and powder [24, 30]. A previous study reported the lowest bleaching rate for the charcoal-containing toothpaste in comparison with blue covarine, hydrogen peroxide, and other abrasives [8]. Palandi et al. [32] found the toothpaste containing activated charcoal was not effective in changing teeth color. The difference between the results of their study and the current one can be attributed to the number of brushing cycles, which was about 824 cycles (equivalent to 14 days) in comparison to 10,000 cycles of this study.

There are concerns about adverse effects of charcoal on resin composite or even ceramic restorations [33]. The dark gray color resulting from charcoal accumulation within the gingival sulcus or margins of restorations may necessitate replacing these materials due to esthetics requirements. In contrast with the present study results, which showed charcoal-containing toothpaste can lighten the pigmented composites through removing surface stains, Torso et al. [10] revealed that the lightness of composite samples submitted to charcoal-based dentifrices (toothpaste and powder) was significantly lower than that of conventional dentifrices.

It has been shown the wear resulting from the abrasive particle of charcoal can compromise the longevity of restorations and should not be neglected. The starry and angular shape of charcoal particles would effectively increase the wear rate of charcoal-containing toothpaste [17]. Due to limitations of this study, although the wear was not investigated, the hardness which was related to wear was evaluated. To enhance the accuracy of the microhardness test, the surface of the samples was abraded with silicon carbide papers to remove the surface grooves. Although both toothpastes generally boosted the surface microhardness of the bovine enamel, it was significantly higher after applying the charcoal-containing toothpaste. This can be related to the presence of 1,450 ppm fluoride in the composition of the evaluated charcoal-containing toothpaste, which recovers the hardness of enamel surface [24]. Since fluoride cannot deposit on the composite surface on the way it occurs on enamel surfaces, the benefits of fluoride did not show on composite specimens. Indeed, the microhardness diminished after application of both types of toothpaste in composite specimens but the difference was not noticeable. Concurring with the present study results, Farghal and Elkafrawy [9] showed no significant difference between control group (distilled water) and charcoal-containing toothpaste (Perfect White Black) in both microfilled (Heliomolar) and nanohybrid (Tg-nanohybrid) composite resins. Although higher wear and diminished hardness were reported as the side effects of using the activated charcoal [7, 9, 29], this study could not confirm the negative effect of charcoal on enamel hardness.

The results of this study indicated higher surface roughness after applying charcoal toothpaste in the specimens in comparison to noncharcoal toothpaste. The amount of surface roughness in composite specimens was higher than in bovine enamel. However, due to the limited number of samples in each group which is not adequate for statistical analysis, further studies are required. It seems the amount of roughness resulting from using charcoal dentifrices on resin composites may be affected by the filler composition and distribution. In Farghal and Elkafrawy [9] study, both tested composites indicated significant surface roughness after using charcoal-containing toothpaste. However, the microfilled composite presented generally lower surface roughness than nanohybrid composite. This was related to the “organic filler” approach used in the microfilled composite manufacturing process, which made the microfilled composite less susceptible to debonding and elution.

The average relative dentin abrasivity (RDA) of toothpastes was between 60 and 100 [34]. Although the toothpaste with activated charcoal has an RDA close to 50, the size and shape of charcoal particles as well as its composition cause surface roughness [24]. Machla et al. [35] indicated that the abrasiveness of these dentifrices is acceptable in ISO limitations where Colgate MAX WHITE showed an average RDA of 9. Although in most previous studies, greater enamel surface roughness was recorded after using the charcoal-containing toothpastes [9, 17, 32, 36], some authors have found comparable effects of charcoal-based toothpastes on color, roughness and hardness of enamel with regular fluoridated toothpaste [24]. In Dionysopoulos et al. [36] study, the charcoal-containing toothpaste affected the surface morphology of enamel by leaving a heterogeneous surface with numerous large and deep craters plus increased roughness. It has been shown that the charcoal powder can produce surface roughness comparable with bleaching gel containing 10% carbamide peroxide in tooth [30]. The results of Ghajari et al. [28] suggested that the abrasive and whitening effects of three charcoal-containing toothpastes (Bencer, Beverly, and Colgate) on human tooth had no significant difference.

In general, the present study results revealed the beneficial effects of charcoal toothpaste on the removal of surface pigments and the whitening effect on composite resin and teeth surfaces with no adverse effects on surface microhardness. There are some limitations in this *in vitro* study that should be considered when interpreting the obtained results. The application of bovine teeth, insufficient samples for the surface roughness assessment and lack of saliva as a medium of remineralization may be affected the study outcome and necessitate further research. The combination of charcoal and natural compounds such as paraprobiotics, postbiotics, lactoferrin which have been shown promising results on gingival and periodontal health may be interesting topic for future studies [37, 38]. In addition, other features of charcoal-containing toothpastes including biocompatibility [39], fluoride adsorption, antimicrobial properties, longevity, and stability of the whitening should be investigated.

## 5. Conclusion

Based on the findings of this study and considering the limitations of *in vitro* studies:

1. The evaluated charcoal-containing toothpaste significantly improved the overall color and lightness of both enamel as well as resin composite specimens.
2. The application of toothpaste containing active charcoal and fluoride had no adverse effect on the microhardness of tooth enamel and resin composite surface.
3. Charcoal-containing toothpaste enhanced the surface roughness of enamel and resin composites.

## Figures and Tables

**Table 1:** The composition of the evaluated toothpastes of the study according to manufacturers.

Product	Type	Composition	Active agents	Manufacturer
Colgate® Max Fresh Cooling Crystals Toothpaste	Regular toothpaste	Sorbitol, aqua, hydrated silica, sodium lauryl sulfate, aroma, PEG-12, cellulose gum, cocamidopropyl betaine, sodium fluoride (1,450 ppm F¯), sodium saccharin, hydroxypropyl methylcellulose, menthol, limonene, CI 42090, CI 77,891	Hydrated silica	Colgate-Palmolive Company, Piraeus, Greece

Colgate® Max White Charcoal Toothpaste	Whitening toothpaste	Sorbitol, aqua, hydrated silica, sodium lauryl sulfate, aroma, PEG-12, cellulose gum, cocamidopropyl, betaine, sodium fluoride (1,450 ppm F¯), sodium saccharin, tetrapotassium, pyrophosphate, potassium hydroxide, phosphoric acid, xanthan gum, mica, charcoal powder, limonene, CI 77,891	Active charcoal (1% w/w)	Colgate-Palmolive Company, Piraeus, Greece

**Table 2:** Mean and standard deviation (SD), minimum and maximum of color variables after using the toothpastes.

Variables	Specimens	Toothpastes	Mean ± SD	Min	Max	*P* value
*ΔL*	Enamel *N* = 18	Charcoal free (Colgate® Max Fresh)	12.44 ± 1.24	0.47	17.91	0.001^†^
		Charcoal (Colgate® MAX WHITE)	24.56 ± 2.57	5.63	30.21	
	Composite *N* = 18	Charcoal free (Colgate® Max Fresh)	0.67 ± 0.65	−0.31	2.01	0.021^‡^
		Charcoal (Colgate® MAX WHITE)	1.4 ± 1.08	−0.51	3.64	

*Δa*	Enamel *N* = 18	Charcoal free (Colgate® Max Fresh)	−1.77 ± 1.45	−4.28	0.19	0.0001^‡^
		Charcoal (Colgate® MAX WHITE)	−3.86 ± 1.7	−7.69	−1.13	
	Composite *N* = 18	Charcoal free (Colgate® Max Fresh)	−0.29 ± 0.44	−1.08	0.72	0.025^†^
		Charcoal (Colgate® MAX WHITE)	−0.61 ± 0.39	−1.6	−0.2	

*Δb*	Enamel *N* = 18	Charcoal free (Colgate® Max Fresh)	−1.24 ± 1.47	−3.67	1.4	0.009^‡^
		Charcoal (Colgate® MAX WHITE)	−2.95 ± 2.12	−5.83	0.39	
	Composite *N* = 18	Charcoal free (Colgate® Max Fresh)	−0.19 ± 0.33	−0.72	0.4	0.002^‡^
		Charcoal (Colgate® MAX WHITE)	−0.93 ± 0.82	−3.21	0.24	

Statistically significant at *P* < 0.05. ^†^Mann–Whitney *U* test. ^‡^Independent sample *t*-test.

**Table 3:** Mean, standard deviation (SD), minimum and maximum of color change after using the toothpastes.

Variable	Specimens	Toothpastes	Mean ± SD	Min	Max	*P* value
*ΔE*	Enamel *N* = 18	Charcoal free (Colgate® MAX Fresh)	7.49 ± 5.02	0.71	18.23	*P* ≤ 0.001^†^
		Charcoal (Colgate® Max WHITE)	17.83 ± 16.9	5.76	31.4	
	Composite *N* = 18	Charcoal free (Colgate® MAX Fresh)	0.96 ± 0.93	0.15	2.2	*P* ≤ 0.001^‡^
		Charcoal (Colgate® Max WHITE)	1.94 ± 1.2	0.46	5.11	

Statistically significant at *P* < 0.05. ^†^Mann–Whitney *U* test. ^‡^Independent sample *t*-test.

**Table 4:** Mean, standard deviation (SD), minimum and maximum of microhardness values after using the toothpastes.

Variables	Specimens	Toothpastes	Mean ± SD	Min	Max	*P* value
Microhardness	Enamel *N* = 18	Charcoal free (Colgate® Max Fresh)	12.45 ± 51.67	−96.3	105.7	0.04^‡^
		Charcoal (Colgate® MAX WHITE)	49.04 ± 53.19	−77.3	138.3	
	Composite *N* = 18	Charcoal free (Colgate® Max Fresh)	−3.71 ± 3.99	−8.9	7.4	0.23^‡^
		Charcoal (Colgate® MAX WHITE)	−1.3 ± 7.52	−20.9	10.5	

Statistically significant at *P* < 0.05. ^‡^Independent sample *t*-test.

**Table 5:** Mean, standard deviation (SD) of surface roughness after using the toothpastes.

Variable	Specimens	Toothpastes	Mean ± SD
Roughness	Enamel *N* = 2	Charcoal free (Colgate® Max Fresh)	−22.37 ± 17.61
		Charcoal (Colgate® MAX WHITE)	−83 ± 70.81
	Composite *N* = 2	Charcoal free (Colgate® Max Fresh)	−20 ± 26
		Charcoal (Colgate® MAX WHITE)	−83.74 ± 70.81

## Data Availability

The data used to support the findings of this study are available from the corresponding author upon request.
